# Activity of Moxifloxacin Against Biofilms Formed by Clinical Isolates of *Staphylococcus aureus* Differing by Their Resistant or Persister Character to Fluoroquinolones

**DOI:** 10.3389/fmicb.2021.785573

**Published:** 2021-12-15

**Authors:** Tiep K. Nguyen, Frédéric Peyrusson, Wafi Siala, Nhung H. Pham, Hoang A. Nguyen, Paul M. Tulkens, Françoise Van Bambeke

**Affiliations:** ^1^Pharmacologie cellulaire et moléculaire, Louvain Drug Research Institute, Université catholique de Louvain, Brussels, Belgium; ^2^Department of Pharmaceutical Industry, Hanoi University of Pharmacy, Hanoi, Vietnam; ^3^Department of Microbiology, Bach Mai Hospital, Hanoi, Vietnam; ^4^The National Center for Drug Information and Adverse Drug Reactions Monitoring, Hanoi University of Pharmacy, Hanoi, Vietnam

**Keywords:** moxifloxacin, biofilm, resistance, persistence, tolerance, *icaA*, *Staphylococcus aureus*

## Abstract

*Staphylococcus aureus* biofilms are poorly responsive to antibiotics. Underlying reasons include a matrix effect preventing drug access to embedded bacteria, or the presence of dormant bacteria with reduced growth rate. Using 18 clinical isolates previously characterized for their moxifloxacin-resistant and moxifloxacin-persister character in stationary-phase culture, we studied their biofilm production and matrix composition and the anti-biofilm activity of moxifloxacin. Biofilms were grown in microtiter plates and their abundance quantified by crystal violet staining and colony counting; their content in polysaccharides, extracellular DNA and proteins was measured. Moxifloxacin activity was assessed after 24 h of incubation with a broad range of concentrations to establish full concentration-response curves. All clinical isolates produced more biofilm biomass than the reference strain ATCC 25923, the difference being more important for those with high relative persister fractions to moxifloxacin, most of which being also resistant. High biofilm producers expressed *icaA* to higher levels, enriching the matrix in polysaccharides. Moxifloxacin was less potent against biofilms from clinical isolates than from ATCC 25923, especially against moxifloxacin-resistant isolates with high persister fractions, which was ascribed to a lower concentration of moxifloxacin in these biofilms. Time-kill curves in biofilms revealed the presence of a moxifloxacin-tolerant subpopulation, with low multiplication capacity, whatever the persister character of the isolate. Thus, moxifloxacin activity depends on its local concentration in biofilm, which is reduced in most isolates with high-relative persister fractions due to matrix effects, and insufficient to kill resistant isolates due to their high MIC.

## Introduction

*Staphylococcus aureus* is one of the leading causes of hospital-acquired infections. These pathogens are difficult to eradicate, not only because of increasing rates of resistance to entire classes of currently used antistaphylococcal agents, but also due to their capacity to adopt specific lifestyles that make them non-responsive to antibiotics to which they remain susceptible in conventional susceptibility testing, creating a potential for treatment failures, and/or relapses.

Among these lifestyles, biofilms are frequently observed. They are defined as structured consortia of microbial cells (most often adhering on a surface) embedded in a self-produced extracellular matrix mainly made of polysaccharides, extracellular DNA (eDNA), and proteins (Donlan and Costerton, 2002; Hall-Stoodley et al., 2004, 2012). Staphylococci can adhere to both artificial and biological surfaces, allowing them to form strong biofilms in various environments. In patients, biofilms are easily formed at the surface of foreign material, causing prosthetic or catheter infections that are very difficult to treat (Oliveira et al., 2018). Biofilms also grow at the surface of epithelia, causing chronic wounds infections, otitis media, rhinosinusitis, periodontitis, and recurrent tonsillitis, as well as chronic lung infection in patients with cystic fibrosis (Archer et al., 2011; Tong et al., 2015).

In biofilms, bacteria can adopt a transient and non-heritable phenotype defined by the physiological state of biofilm cell populations and leading to antibiotic tolerance (Dincer et al., 2020). Antibiotic tolerance is characterized by reduced bactericidal effects without observable changes in the minimal inhibitory concentrations (MICs) of antibiotics (Brauner et al., 2016). Moreover, in the interior of biofilms where nutrients and oxygen are less abundant, subpopulations of bacteria, which are then defined as antibiotic-induced persisters (Rani et al., 2007; Brauner et al., 2016; Bui et al., 2017) tend to adopt a dormant phenotype, characterized by a slow growth rate.

The mechanisms leading to antibiotic tolerance in biofilms are complex (Olsen, 2015). On the one hand, oxygen gradients and altered metabolism participate to slow growth rate and to the presence of persister cells, which contribute to reduce the antibiotic rate of killing (Conlon et al., 2015; Brauner et al., 2016; Bui et al., 2017). On the other hand, biofilm matrix is an obstacle to the penetration and access of antibiotics to bacteria, explaining their inability to hit deep seated bacteria in these complex structures (Hall and Mah, 2017). Among matrix constituents, polysaccharides are synthesized at the early stages of biofilm development. One of them, poly-*N*-acetyl-glucosamine (PNAG), is produced by the enzymes encoded by the *icaADBC* operon (Heilmann et al., 1996; O’Gara, 2007), which is expressed by the vast majority of *S. aureus* clinical isolates (Cramton et al., 1999). PNAG is considered as a main driver of biofilm formation (Freitas et al., 2018) and plays an important role in their structural integrity *in-vitro* and *in-vivo* (Lister and Horswill, 2014).

At this stage, we still lack of data that systematically examine the respective roles of antibiotic resistance, of persister cells, and of the biofilm matrix in the poor response of staphylococci to antibiotics in biofilm-related infections. In the present study, we used a collection of *S. aureus* assembled in a tertiary hospital in Hanoi, Vietnam (Nguyen et al., 2020a) from samples of patients suffering from persistent or recurrent infections (defined as infections that did not resolved after 5 days of treatment with an antibiotic to which the initial isolate was susceptible, or infections that reactivated a few days after improvement of the patient’s conditions and interruption of the antibiotic treatment, respectively). All isolates were thereafter characterized in Belgium with respect to their resistance profile to commonly used antibiotics (Nguyen et al., 2020a) and to their persister character [as determined against stationary phase cultures exposed to moxifloxacin as reporter antibiotic (Nguyen et al., 2020b)]. Importantly, persistence and resistance were closely related in this collection, with all resistant isolates showing high relative persister fractions (RPF) to moxifloxacin, while only a small proportion of susceptible isolates were high persisters. In addition, susceptible high persisters also acquire resistance to moxifloxacin more rapidly than susceptible isolates with low RPF (Nguyen et al., 2020b). This indicates that persistence may promote evolution toward resistance, as previously documented for *Escherichia coli* (Barrett et al., 2019; Windels et al., 2019), and therefore links to some extent these two properties.

We selected an equivalent number of non-clonal moxifloxacin-susceptible and -resistant isolates in this collection (all belonging to Agr group I; Nguyen et al., 2020a), taking also into account their relative persister fraction to moxifloxacin (Nguyen et al., 2020b). We then determined their capacity to form biofilm as well as the global composition of the biofilm matrix. We also studied the activity of moxifloxacin against preformed biofilms. Moxifloxacin is a fluoroquinolone endowed with strong bactericidal activity, and with indications and large clinical use as antistaphylococcal agent. It was selected here because, in the same model, it proved to be one of the most active drugs against biofilms formed by the susceptible, reference strain ATCC 25923 used here as a control (Bauer et al., 2013), allowing to clearly document a reduction in activity related to resistance or persistence.

We show that isolates with a high proportion of persister cells in stationary-phase culture, which also include a majority of resistant isolates, produce more biofilm (enriched in PNAG) than those with a lower proportion of persister cells, and that moxifloxacin activity against biofilms is reduced in proportion of the amount of biomass produced, essentially against resistant isolates, for which the concentration of the drug in the biofilm remains below their MIC.

## Materials and Methods

### Main Products and Reagents

Moxifloxacin HCl (microbiological standard; potency: 90.9%) was provided by Bayer HealthCare (Leverkusen, Germany). Resazurin sodium salt, crystal violet solution, calcofluor white (CFW), proteinase K, and cation-adjusted Muller Hinton broth 2 (CA-MHB) were obtained from Sigma-Aldrich (St-Louis, MO); Tryptic Soy Broth (TSB), Tryptic Soy Agar (TSA), and multiwell plates, from VWR (Radnor, PA); bovine serum albumin, from Thermo Fisher Scientific Rockford, IL); 5-cyano-2, 3-ditolyl tetrazolium chloride (CTC) (RedoxSensor vitality kit), from Invitrogen (Carlsbad, CA); and Quick Start™ Bradford Protein Assay, from BioRad (Hercules, CA).

### Bacterial Strains and Their Characterization

The methicillin-susceptible *S. aureus* (MSSA) ATCC 25923 and the methicillin-resistant *S. aureus* (MRSA) ATCC 33591 were used as references. Two strains expressing GFP under the control of a tetracycline-inducible promoter (RN4220-palc and 1214-palc, Nguyen et al., 2020b), were used in specific experiments. In addition, 18 clinical, non-isogenic, isolates of *S. aureus* collected from patients with persistent or recurrent infections at the Bach Mai hospital, Hanoi, Vietnam, were included in the study. The patients were hospitalized in urology or rheumatology, and the collected samples included mainly infected catheters, pus, tophi, abscesses, or blood. These isolates were previously characterized (Supplementary Table 1) by molecular *spa* and *agr* typing; their MRSA character was established by measuring cefoxitine MIC and detecting *mecA* and *mecC* by PCR (Nguyen et al., 2020a). The MIC of moxifloxacin was previously determined (Nguyen et al., 2020b) by broth microdilution following CLSI recommendations (Clinical and Laboratory Standards Institute, 2020) and susceptibility breakpoints were those established by EUCAST;^1^ the relative persister fraction was previously established (Nguyen et al., 2020b) by measuring the number of CFU after 5 h incubation of a stationary phase culture with moxifloxacin at 100 times its MIC, according to a published procedure. In this method, the persister character of each isolate was evaluated by its persister fraction relative to that of a reference (De Groote et al., 2009), here ATCC 25923, to allow for a more accurate comparison of persister fractions determined in independent experiments. The relative persister fraction of ATCC 33591 is 1 (same as that of ATCC 25923), so that this strain was used as a reference rather than ATCC 25923 in specific experiments because it forms a biofilm more comparable to clinical isolates in terms of CFUs counts (see Supplementary Figure 1). Isolates were categorized in three groups according to their persister and resistant character (Nguyen et al., 2020b), respectively, referred to as susceptible with low relative persister fraction [S-LP; MICs ≤ 0.25 mg/L; persister fraction relative to ATCC 25923 ≤ 10 (6 isolates)], as susceptible with high relative persister fraction [S-HP; MICs ≤ 0.25 mg/L; persister fraction relative to ATCC 25923 > 10 (3 isolates)], and resistant with high relative persister fraction [R-HP; MICs > 0.25 mg/L; persister fraction relative to ATCC 25923 > 10 (9 isolates)]. Metabolic activity was assessed by following the capacity of each isolate to metabolize the non-fluorescent substrate resazurin in fluorescent resorufin (λ_*exc*_ 560 nm/λ_*em*_ 590 nm) (Bauer et al., 2013). Planktonic cultures at different OD_620 *nm*_ were incubated with resazurin (10 mg/L in PBS) for up to 60 min and fluorescence was measured over time in a M3 SPECTRAmax microplate spectrofluorometer (Molecular Devices LLC, Sunnyvale, CA).

### Biofilm Model

Biofilms were obtained using as a starting inoculum bacteria transferred from frozen stocks onto TSA and incubated overnight at 37°C, after which 10 colonies were inoculated in TGN (TSB supplemented with 2% NaCl and 1% glucose), and the bacterial density of the starting inoculum was adjusted to an OD_620 *nm*_ of 0.005 (approx. 10^7^ CFU/mL). Biofilms were grown in 96-well plates at 37°C for 24 h (Bauer et al., 2013), after which the medium was renewed (controls) or replaced by a medium containing moxifloxacin for 24 h.

### Quantification of Biofilms and of Their Matrix Constituents

These analyses were performed on control biofilms after 2 successive cycles of 24 h of growth with mid-period renewal of the medium as described above. Biofilm biomass was evaluated by measuring the absorbance of crystal violet at 570 nm using a M3 SPECTRAmax microplate spectrofluorimeter as previously described (Bauer et al., 2013), except that we used 200 μL of crystal violet (Sigma-Aldrich) at 10% (V/V, final concentration 2.3 g/L) in water to stain the dry biofilms for 15 min. Bacterial viability was determined by CFU counting (Nguyen et al., 2020a). Biofilms were gently washed twice with sterile phosphate buffer saline (PBS), collected in sterile water, and sonicated 5 min using a sonication bath to insure the liberation of bacteria from the matrix. After appropriate dilution in PBS, aliquots were spread on TSA and CFU were counted after overnight culture.

To obtain a gross estimation of their polysaccharide content, biofilms were grown in 96-well plates, the medium was removed and wells were washed twice with PBS. Biofilms were fixed by heat at 60°C for about 2 h (Bauer et al., 2013). Polysaccharides were then stained by Calcofluor White (CFW), a fluorophore that preferentially binds to β-1,3 and β-1,4 polysaccharides (Maeda and Ishida, 1967), using a procedure based on that of Stiefel et al. (2016). In brief, 200 μL of CFW (0.25 mg/mL in H_2_O) was added in each well and incubated during 15 min in the dark, after which the staining solution was removed, and the plates were washed twice with PBS. Two hundreds μL of absolute EtOH were added per well and incubated for 5 min in the dark to resolubilize the dye, after which fluorescence was read (λ_*exc*_ 360 nm/λ_*em*_ 460 nm) in a M3 SPECTRAmax microplate spectrofluorometer.

To quantify proteins, biofilms formed in 96-well plates were washed thrice with sterile PBS and detached with 200 μL cold sterile distilled water by pipetting. Proteins were quantified using the Quick Start Bradford Protein Assay according to the manufacturer’s instructions, except that 50 μL of samples were mixed with 200 μL of reagent. After 15 min incubation in the dark under agitation at 100 rpm, absorbance was read at 595 nm, using a SPECTRAmax microplate spectrofluorometer and protein content was calculated using a standard curve obtained with bovine serum albumin.

To quantify extracellular DNA (eDNA), biofilms were grown in 4 mL TGN in 6-well plates, then washed thrice with PBS, detached with 1 mL cold sterile distilled water by rapid pipetting. eDNA was purified by a method adapted from Kreth et al. (2009). Briefly, samples were incubated at 37°C for 1 h in the presence of 5 μg/mL proteinase K and centrifuged at 16,000 *g* for 2 min. The supernatant was collected and eDNA extracted with phenol:chloroform:isoamyl alcohol (25:24:1). Samples were centrifuged at 16,000 *g* for 5 min, and 800 μL of aqueous phase were collected. eDNA was precipitated by the addition of 500 μL isopropanol and 80 μL 3 M sodium acetate, pelleted by centrifugation at 16,000 *g* for 10 min, air-dried, and re-suspended in 50 μL nuclease-free H_2_O. The purity of DNA in each sample was checked on agarose gels and its concentration determined using a NanoDrop™ spectrophotometer (Thermo Fisher Scientific; Waltham, MA).

### Moxifloxacin Activity Against Bacterial Biofilms

Moxifloxacin activity was determined against 24-h biofilms, as previously described (Bauer et al., 2013). In brief, the culture medium was removed and replaced by a control medium or a medium containing moxifloxacin at increasing concentrations (0.001–1,000 mg/L). Biofilms were reincubated for 24 h at 37°C. Remaining biomass was quantified by crystal violet staining, and viability, by CFU counting using the same procedures as for control biofilms. Data were plotted as concentration-response curves and used to fit Hill equations, which allowed us to calculate pharmacodynamic parameters, like the maximal efficacy (E_*max*_; i.e., maximal reduction in CFU or crystal violet absorbance as extrapolated for an infinitively large antibiotic concentration), and relative potency, estimated by the calculation of C_–1l*og*_ (i.e., concentration needed to reduce of 1 log_10_ CFU the bacterial counts in biofilms) or of C_20%_ (i.e., concentration needed to reduce of 20% crystal violet absorbance).

### Expression of *icaA*

The presence of the *ica* locus in clinical isolates was checked by PCR. Genomic DNA was extracted from overnight cultures in CA-MHB using the DNeasy Blood and Tissue Kit (Qiagen, Hilden, Germany) following the manufacturer’s instructions. The purity of DNA in each sample was checked on agarose gels. *icaA* was amplified by PCR using 1 μL of DNA, 2.5 μL of 10x DreamTaq Buffer, 0.8 μL of 10 mM dNTPs, 1 μL of 10 mM of each primer (*icaA-F* :CGAGAAAAAGAATATGGCTG; *icaA-R:* ACCATGTTGCGTAACCACCT), 0.125 μL DreamTaq DNA polymerase (Thermo Fisher Scientific, Waltham, MA), and 18.58 μL sterile nuclease-free water (Thermo Fisher Scientific) and the following PCR program: denaturation at 98°C for 1 min, 30 amplification cycles (98°C for 10 s, 60°C for 30 s, and 72°C for 60 s), and a final cycle of 72°C for 10 min. PCR products were visualized on agarose gel. As *icaA* was detected in all isolates, the expression of the gene was then quantified by quantitative real-time PCR (q-RT-PCR). To this effect, RNAs were isolated from 3 h-old biofilms grown in 6-well polystyrene plates (4 mL of culture at OD_620 *nm*_ = 0.005). Biofilms were collected by pipetting, pelleted by centrifugation at 4,000 rpm for 7 min, washed twice in sterile phosphate buffered saline (PBS) and pelleted again. Cell pellets were resuspended in 100 μL of solution containing 270 μg lysozyme and 10 μg lysostaphin (Sigma) and incubated 30 min at room temperature to achieve bacterial lysis. Total RNA was isolated using The InviTrap^®^ Spin Universal RNA Mini Kit (Stractec, Berlin, Germany). Samples were cleaned from DNA using the TURBO DNA-free™ Kit (Thermo Fisher Scientific). RNA quality and quantity were checked by measuring A_260 *nm*_ and A_280 *nm*_ using a NanoDrop spectrophotometer. Purified RNA was converted to cDNA using the transcription first strand cDNA synthesis kit (Roche Applied Science) with random hexamer primers according to the manufacturer’s instructions, and then diluted 10 times. Quantitative PCR reactions were performed in triplicates in 96-well plates (Greiner) using 5 μL of cDNA, 12.5 μL of SYBR Green Master Mix (Bio-Rad), 2 μL of 5 mM of each primer, and 3.5 μL of sterile RNase-free water (Ambion). *gmk* gene (encoding a guanylate kinase) was used as an housekeeping gene and amplified with following primers: *gmk-F:* TCAGGACCATCTGGAGTAGGTAAAG; *gmk-R*: TTCACGCATTTGACGTGTTG. For *icaA*, we used the same primers as those described above for PCR amplification of gene and the following program: denaturation at 95°C for 3 min, 40 amplification cycles at 95°C for 15 s and 60°C for 60 s. A melting curve was run at the end of the PCR cycles to check for the presence of a unique PCR reaction product. Relative expression levels of *icaA* were calculated using the ΔΔCt method, with *gmk* expression levels as a control and compared with the expression level measured in the reference strain ATCC 33591.

### Confocal Microscopy for Determination of Moxifloxacin Penetration Within Biofilms

According to a previously described procedure (Siala et al., 2014), 24 h biofilms were grown on coverslips, incubated during 1 h with 20 mg/L moxifloxacin, washed twice with 1 mL PBS and stained for 30 min in the dark with 1 mL 0.5 mM CTC, a colorless, non-fluorescent and membrane-permeable compound, which is reduced by viable bacteria to fluorescent, insoluble CTC-formazan (Kim et al., 2008). Stained biofilms were then washed with 1 mL PBS and then examined in a Cell Observer^®^ SD confocal fluorescent microscope (Carl Zeiss AG, Oberkochen, Germany) using spinning disc technology (Yokogawa Electric Corporation, Tokyo, Japan) and controlled by the AxioVision software (AxioVs40 V 4.8.2.0; Zeiss). Excitation/emission wavelengths were set at 415 nm/500–550 nm for moxifloxacin and 488 nm/570–620 nm for CTC-formazan. Moxifloxacin concentrations within biofilms were then calculated using calibration curves built using moxifloxacin solutions at concentrations ranging from 5 to 50 mg/L and examined in the microscope using the same settings as for samples.

### Flow Cytometry

We used two clinical isolates with susceptible low-persister (RN4220) and susceptible high-persister (1,214) phenotype, respectively, which were transformed by the pALC2084 to express Green fluorescent protein (GFP) under a xyl/tetO inducible promoter (Nguyen et al., 2020b). They were used to form biofilm as described above except that they were maintained in the presence of 125 ng/mL tetracycline (to induce GFP production) and 10 μg/mL chloramphenicol (as a selecting agent) during the 24 h of biofilm growth. They were then incubated with moxifloxacin in the absence of tetracycline and chloramphenicol during 24 h, after which bacteria were isolated from biofilms as described above and used for CFUs counting and flow cytometry analysis as previously described (Nguyen et al., 2020b) using a FACSVerse cytometer (BD Biosciences) and the FlowJo 10.5.2 software (TreeStar Inc., Ashland, OR) for data analysis.

## Results

### Biofilm Formation by Clinical Isolates

**FIGURE 1:**
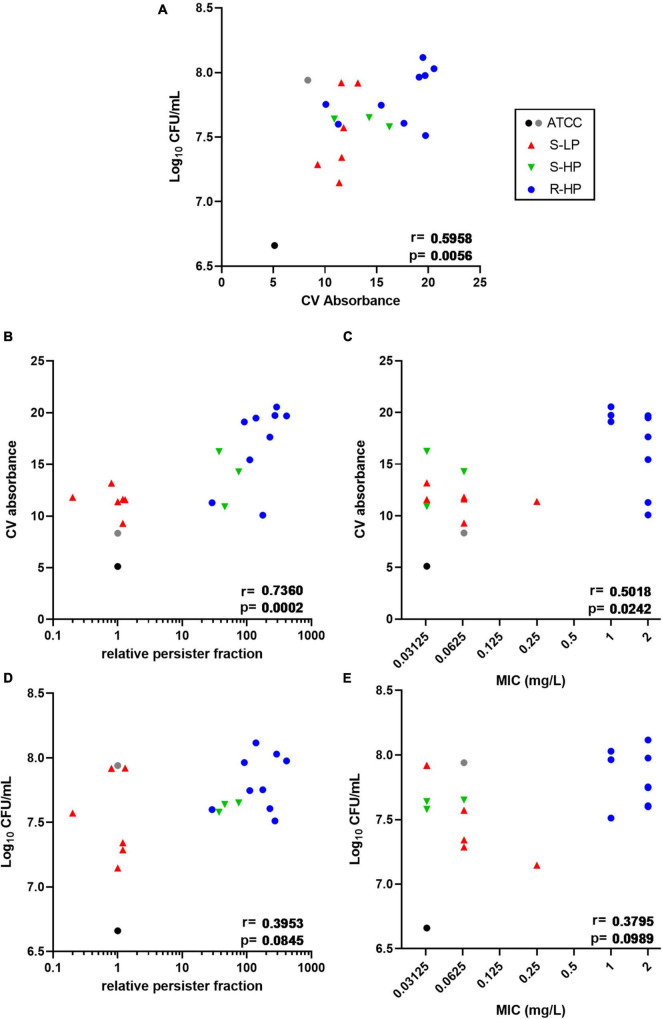
Quantification of biofilms (biomass *via* crystal violet (CV) absorbance and CFUs counts) made by clinical isolates and reference strains ATCC 25923 (black dot) and ATCC 33591 (gray dot) after 48 h of incubation in TGN (with renewal of the medium at 24 h). Symbols for clinical isolates depend on their resistant or persister phenotype [S-LP (red): susceptible, with low relative persister fraction; S-HP (green) susceptible, with high relative persister fraction; R-HP (blue): resistant, with high relative persister fraction]. Data are shown as mean of 6 independent experiments for the references, and of 2–3 independent experiments for each isolate. Correlations between CFUs counts and crystal violet absorbance **(A)**, CV absorbance and relative persister fraction or MIC of each isolate **(B,C)**, and between CFU counts and relative persister fraction or MIC of each isolate **(D,E)**. Statistical analysis: r (correction coefficient) and *p*-values for the correlation.

In a first step we compared 18 clinical isolates (9 susceptible and 9 resistant to moxifloxacin) and the reference strain ATCC 25923 for biofilm formation after 48 h of incubation with renewal of the medium at 24 h. Bacterial survival was first assessed by measuring their metabolic activity toward the non-fluorescent dye resazurin (reduction into fluorescent resorufin). However, since a large proportion of the clinical isolates showed a high relative persister fraction, which, by definition, implies a decreased metabolic activity, we checked in preliminary experiments whether this method could be used to correctly quantify the amount of living bacteria in biofilms. As shown in Supplementary Figure 2, the fluorescence signal generated by the clinical isolates was not only often lower than that of ATCC 25923, but was also highly variable among isolates, irrespective of the proportion of persisters they could generate. Thus, the method was found unusable for quantitative determination of viable bacteria in our experimental setting, as already reported by others in similar experiments (Sandberg et al., 2009; Alonso et al., 2017). Bacterial viability was therefore evaluated using CFU counting, while biomass was measured using crystal violet staining. Supplementary Figure 1 shows that all clinical isolates were high biofilm producers, with crystal violet absorbance values being globally 2 or 3 times higher than for the two reference strains (ATCC 33591 and ATCC 25923) (Supplementary Figure 1A), and CFU counts reaching values 10-fold (1 log_10_) higher than for ATCC 25923, but similar to those recovered from ATCC 33591 biofilms (Supplementary Figure 1B). An in-depth analysis of biofilm formation by clinical isolates depending on their persister or resistant phenotype is presented in Figure 1, with reference strains added as internal controls. A significant correlation was observed between biomass and CFU counts (Figure 1A). We then examined the correlation between biomass or CFU counts in the biofilms and the relative persister fraction (RPF) of each isolate (Nguyen et al., 2020b) or the MIC of moxifloxacin. A highly significant correlation was noticed between biomass and RPF (Figure 1B), and to a lower extent, between biomass and MIC (Figure 1C). In contrast, the correlations between CFU and the two same characteristics of the isolates did not reach significance (Figures 1D,E).

### Analysis of Matrix Composition in Clinical Isolates

**FIGURE 2:**
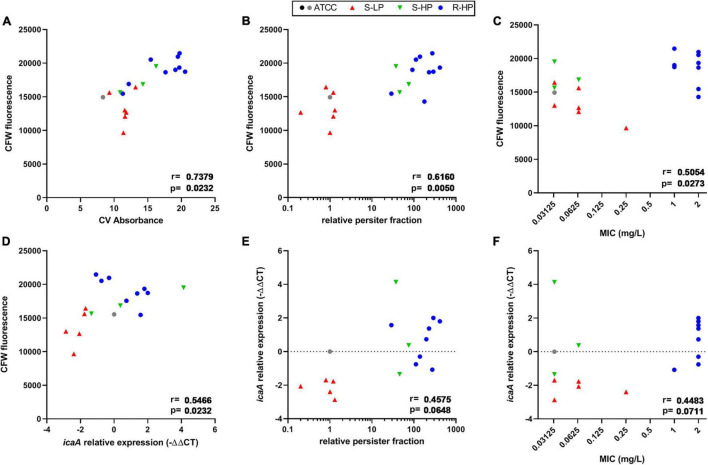
Quantification of polysaccharide content and *icaA* expression in biofilms made by clinical isolates and as compared to reference strains ATCC 25923 (black dot) and ATCC 33591 (gray dot). Symbols for clinical isolates depend on their resistant or persister phenotype [S-LP (red): susceptible, with low relative persister fraction; S-HP (green) susceptible, with high relative persister fraction; R-HP (blue): resistant, with high relative persister fraction]. **(A,D)** Show the correlation between CFW (calcofluor white) fluorescence as a surrogate for β-1,3 and β-1,4 polysaccharides in 48 h-biofilms and crystal violet absorbance at 48 h or *icaA* expression at 3 h, respectively. **(B,C,E,F)** Show correlations between CFW fluorescence or *icaA* expression and the relative persister fraction or the MIC of each isolate, respectively. Statistical analysis: r (correction coefficient) and *p*-values.

In order to better characterize the differences in biomass production among isolates, we analyzed their main matrix constituents in the same conditions. A strong correlation was found between crystal violet absorbance and fluorescence of calcofluor-white (CFW), which binds to β-1,3 and β-1,4 polysaccharides, including PNAG (Figure 2A), but not with the protein or eDNA contents in the biofilms (Supplementary Figure 3). The CFW signal was also significantly correlated with the RFP of clinical isolates, and to a lesser extent, with the moxifloxacin MIC (Figures 2B,C). Since poly-*N*-glucosamine is synthesized by enzymes encoded by the *icaADBC* operon, we quantified the expression of *icaA* for all clinical isolates after 3 h of incubation, knowing that the expression of this gene is upregulated mainly during the early stages of biofilm development (Kot et al., 2018; Idrees et al., 2021). These expression levels were then compared to that of ATCC 33591, since this reference strain produced a biofilm containing a similar amount of viable bacteria as the clinical isolates (see Supplementary Figure 1B) and the same persister fraction as ATCC 25923 (RPF = 1). A significant correlation between *icaA* expression and CFW fluorescence was noticed (Figure 2D); *p*-values were slightly > 0.05 for correlations between *icaA* expression and the RPF of the isolates or moxifloxacin MIC (Figures 2E,F).

### Activity of Moxifloxacin Against Biofilms

**FIGURE 3:**
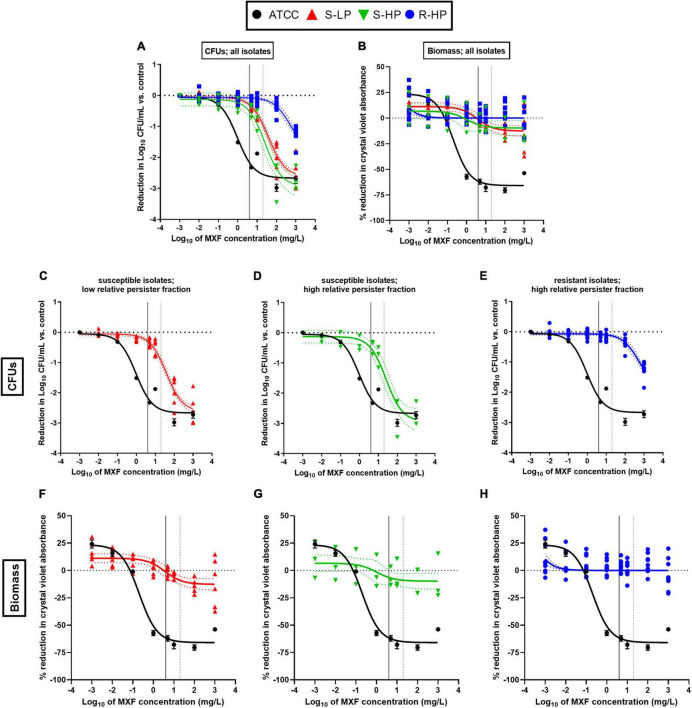
Concentration-response curves of the activity of moxifloxacin (MXF) against 24h-biofilms formed by clinical isolates as compared to a reference strain (ATCC 25923), after 24 h of incubation with the antibiotic over a broad range of concentrations. **(A,C–E)** Reduction in CFU numbers as compared to controls (non-exposed to antibiotic); **(B,F–H)** reduction in biomass (assessed by crystal violet absorbance) in percentage of controls. The data were used to fit a Hill quadratic function with slope factor set to 1 (sigmoidal function) for ATCC 25923 (black lines) and pooled values from all clinical isolates (colored lines) except for biomass in biofilms formed from resistant isolates with high relative persister fractions **(H)** for which a simpler logarithmic dose-response function was used since fitting a Hill function gave rise to ambiguous results. Data are shown as means and SD (*n* = 6) for ATCC 25923 and as mean (*n* = 2) for each individual isolate. The dotted lines above and below the regression line show the 95% confidence interval. **(A,B)** All isolates; **(C,F)** susceptible isolates with low relative persister fraction (S-LP); **(D,G)** susceptible isolates with high relative persister fraction (S-HP); **(E,H)** resistant isolates with high relative persister fraction (R-HP). The curve for ATCC 25923 is repeated in all panels to facilitate comparison with these isolates. The black vertical line on the graphs corresponds to the human C_*max*_ of moxifloxacin (4 mg/L) and the gray dotted vertical line, to the concentration used for confocal microscopy studies (Figure 4).

We then evaluated the effects exerted by moxifloxacin on bacterial viability and biomass in biofilms formed by clinical isolates in comparison with ATCC 25923 and 33591. To this effect, 24 h-old biofilms were exposed to this antibiotic over 24 h, using a broad range of concentrations in order to study the entire concentration-effect relationships (Figures 3A,B). The insure graph readability, isolates were also stratified here according to their phenotype [susceptible with low RPF (S-LP); susceptible with high RPF (S-HP), or resistant with high RPF (R-HP)] (Figures 3C–H). Except for biomass in biofilms developed by resistant isolates (R-HP), a quadratic Hill function with slope factor set to 1 could be fitted to all data (Figure 3 and Table 1 for the definition and values of the corresponding pertinent pharmacodynamic parameters used in our analysis). Considering ATCC 25923 first, moxifloxacin caused a concentration-dependent decrease in both CFUs and biomass, with EC_50_ (i.e., inflection point of the Hill curve) and C_–1l*og*_ or C_20%_ values (indicators of the relative potency) being both at low multiples of the moxifloxacin MIC. At high concentrations, the maximal reduction in viability (E_*max*_) reached −2.7 log_10_ CFU and biomass was reduced to 34% of its control value (biofilms incubated in the absence of antibiotic). Against ATCC 33591 (Table 1), moxifloxacin was as effective (similar E_*max*_) as against ATCC 25923 regarding CFUs but less against biomass; it was also was considerably less potent for both criteria (higher C_–1l*og*_ and C_20%_ values). For susceptible clinical isolates, and focusing first on the effect exerted by moxifloxacin on viability (Figures 3C,D), the relative maximal efficacy was almost reached at the highest concentration tested and not different between S-LP and S-HP subpopulations [E_*max*_: −2.6 to −2.9 log_10_ CFU for S-LP and S-HP isolates, respectively, see also Supplementary Figure 4 and Supplementary Table 2 where data are not normalized as difference from the control (not exposed the antibiotic), illustrating the fact that the actual counts of CFU are higher in clinical isolates]. In contrast, the relative potency of moxifloxacin was markedly decreased since 28–46-fold larger concentrations than for ATCC 25923 were needed to decrease the CFU counts to 10% (1 log_10_ decrease) of their original value, with again no significant difference between susceptible isolates with low (S-LP) or high (S-HP) RFP. For resistant isolates with high RFP (R-HP), the relative potency of moxifloxacin was low, so that only the upper part of the “best-fit” Hill function could be documented by actual data points, making the determination of the maximal relative efficacy value uncertain (Figure 3E). Considering the effect of moxifloxacin on biomass (Figures 3B,F–H), only minor reductions were seen for biofilms formed by susceptible isolates, and no significant effect for biofilms formed by resistant isolates. Restricting the analyses to changes observed at a moxifloxacin concentration corresponding to its average human C_*max*_ [4 mg/L (Sullivan et al., 1999); see the black thin vertical line on the graphs], the antibiotic caused only marginal decreases in bacterial counts (less than 0.5 log_10_ CFU decrease against susceptible isolates and no reduction against resistant isolates) and no effect on biomass in all cases.

**TABLE 1:** Pharmacodynamic parameters for concentration-response curves shown in Figure 3.

Strains	Viability			Biomass		
	E_*max*_^a^ Δ log_10_ CFU/mL from control	EC_50_^b^ mg/L (CI)	C_–1l*og*_^c^ mg/L (× MIC)	E_*max*_^a^ % reduction vs. control	EC_50_^b^ mg/L (CI)	C_20%_^c^ mg/L (× MIC)
ATCC 25923	−2.7 ± 0.2 (A)	0.9 (0.3–3.5) (A)	0.5 (16×)	−66 ± 2 (A)	0.2 (0.1–0.3) (A)	0.06 (2×)
ATCC 33591	−2.9 ± 0.2 (A)	11.5 (7.1–18.5) (B)	20.9 (653×)	−43 ± 3 (B)	3.9 (1.8–7.8) (B)	4.27 (133×)
Susceptible, low relative persister fraction (S-LP)	−2.6 ± 0.1 (A)	38.2 (26.0–57.4) (C)	14.0 (56–438×)	−13 ± 3 (C)	3.7 (0.5–12.7) (B)	>1,000 (>500×)
Susceptible, high relative persister fraction (S-HP)	−2.9 ± 0.2 (A)	25.4 (12.6–51.5) (C)	23.0 (358–716×)	−10 ± 4 (C)	1 (0.003–40) (B)	>1,000 (>500×)
Resistant, high relative persister fraction (R-HP)	−1.9 ± 0.2 ^d^ (C)	529.2 (288–15,201) (D)	562 (231–562×)	NA^e^	NA^e^	>1,000 (>500×)

*Pharmacodynamic parameters calculated based on a downward Hill-Langmuir function fitted to the data changes in the number of viable bacteria (CFU/mL; in log_10_ units) or of crystal violet absorbance (% control value) compared to an untreated 24 h-biofilm both after 24 h of incubation at 37°C.*

*^a^Maximal relative efficacy (decrease in signal for an infinitely high concentration of moxifloxacin).*

*^b^Moxifloxacin concentration for which the effect is halfway between E_min_ (change of signal for an infinitely low concentration of moxifloxacin) and E_max_ (defined in note^a^).*

*^c^Moxifloxacin concentration for which a 1 log_10_ CFU/mL decrease or a reduction of 20% in crystal violet absorbance is measured.*

*^d^Uncertain value because the fitting of the downward Hill-Langmuir function based on data covered only the upper part of the curve.*

*^e^Not applicable (Hill equation cannot be fitted to the data). Statistical analysis: comparison between groups of strains for each parameter (one-way ANOVA with Tukey post-hoc test): data with different letters are significantly different from one another (p < 0.05). For EC_50_ values, statistics were performed on log_10_ values (symmetrically distributed). Data are shown as means and SD (E_max_) or as mean and 95% confidence interval (EC_50_).*

### Concentration of Moxifloxacin in Biofilms

**FIGURE 4:**
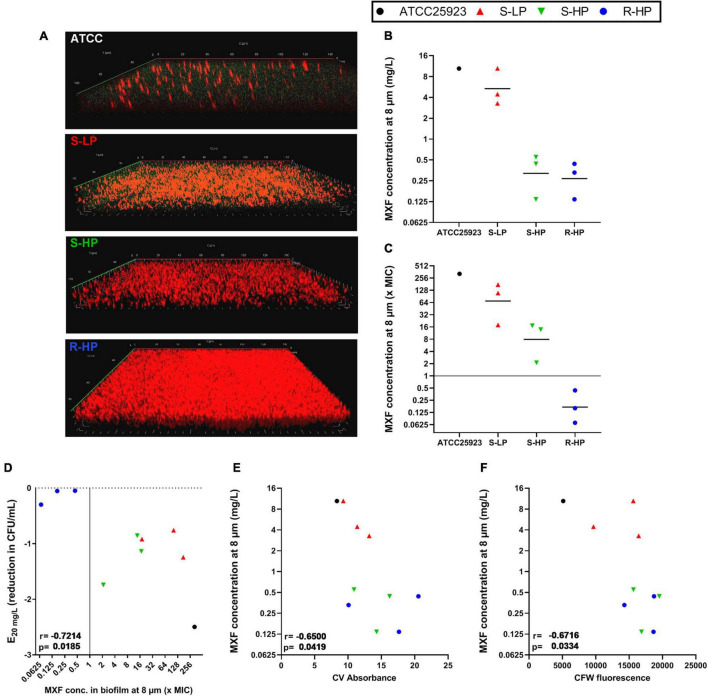
Concentration of moxifloxacin in biofilms of ATCC 25923 or of three selected isolates from each of the three phenotypic groups [susceptible with low relative persister fraction (S-LP; red); susceptible with high relative persister fraction (S-HP; green), resistant with high relative persister fraction (R-HP; blue)] as measured at a deepness of 8 μm (approx. half of the biofilm thickness for clinical isolates) in the corresponding biofilm. **(A)** The pictures show a representative confocal image for 1 isolate in each subgroup that was used to calculate antibiotic concentrations. Biofilms were incubated during 1 h with 20 mg/L moxifloxacin (green signal) and 0.5 mM cyano-2,3-ditolyl tetrazolium chloride [CTC; detecting viable bacteria through its metabolic conversion into fluorescent formazan dye by living bacteria (red signal)]. **(B,C)** Concentration expressed in mg/L or in multiple of the MIC of the respective isolate with the black horizontal line corresponding to the geometric mean value. **(D)** Correlation between efficacy at 20 mg/L and moxifloxacin concentration at 8 μm. **(E,F)** Correlation between moxifloxacin concentration at 8 μm and biomass or CFW fluorescence of the corresponding biofilm (data from Figures 1, 2). The plain horizontal **(C)** or vertical **(D)** line corresponds to the MIC. Statistical analysis: r (correction coefficient) and *p*-values.

In a next step, we measured the concentrations reached by moxifloxacin in the deepness of biofilms, again comparing those formed by isolates belonging to each the three phenotypic groups to that formed by ATCC 25923. To this effect, biofilms were examined in confocal microscopy, taking advantage of the intrinsic fluorescence of moxifloxacin, and using the reduction of 5-cyano-2,3-ditolyl tetrazolium chloride (CTC) to formazan to detect living, metabolically-active, bacteria (Figure 4A). Biofilms were exposed to a concentration of 20 mg/L moxifloxacin to allow for its reproducible detection by fluorescence in the biofilm (Siala et al., 2014). Moxifloxacin concentration was high in biofilms from the reference strain and from S-LP isolates, reaching values well above the MIC of these isolates. Moxifloxacin concentration was lower in biofilms from isolates with high RPF, but still above the MIC for S-HP isolates, which was no more the case for R-HP isolates (Figures 4B,C).

Using these data together with those from Figures 1, 3, we then examined the relationships between moxifloxacin antibacterial activity in the biofilm (reduction of CFU at 20 mg/L) and its concentration in the biofilm. We observed a significant correlation between these two parameters (Figure 4D). In addition, the concentration of moxifloxacin in the biofilm was significantly inversely correlated with the biomass of each individual biofilm, and even more with the fluorescence of CFW (Figures 4E,F).

### Moxifloxacin Kill Rate and Bacterial Division in Biofilms

The persister character of the isolates had been determined in stationary phase cultures. We therefore wondered whether this persister character was maintained when bacteria were encased into biofilms and exposed to antibiotic pressure in this environment. As persisters are characterized by a non-dividing status (Roostalu et al., 2008), we followed bacterial multiplication within the biofilm, using previously constructed transformants of a S-LP strain (RN4220) and of a S-HP strain (1214) having the same susceptibility to moxifloxacin (MIC, 0.064 mg/L) and expressing GFP under the control of a tetracycline-inducible promotor (Nguyen et al., 2020b). These two strains also produce similar biofilms regarding biomass as well as CFU counts in control conditions (10^7.3–7.4^ CFU/mL, both in the presence or in the absence of tetracycline; CV absorbance: 6.7–6.9 or 6.1–6.3 in the absence of or in the presence of tetracycline, respectively).

**FIGURE 5:**
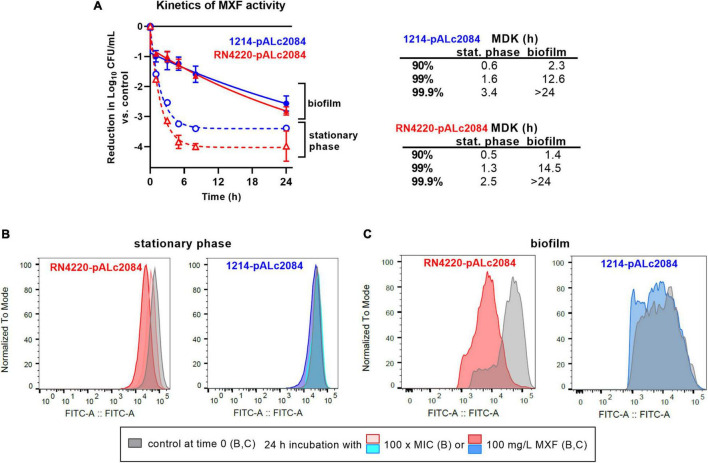
Tolerance and persistence in biofilms. **(A)** Kill curve of moxifloxacin (100 mg/L = 1666 × MIC) against stationary phase cultures (dotted lines) or biofilms (plain lines) from strains RN4220 (S-LP) and 1214 (S-HP) transformed by the pALc2084 plasmid to inducibly express GFP. Data are mean ± SD of triplicates. The adjacent table shows MDK (minimum duration for killing) calculated for reductions of 1, 2, or 3 log_10_ CFU from the initial inoculum, based on the corresponding one phase (*R*^2^ > 0.992; stationary phase culture) or two phases (*R*^2^ > 0.997; biofilm) exponential decays regressions. **(B,C)** Flow cytometric profiles of bacteria collected at time 0 or 24 h in the same conditions. **(B)** Bacteria recovered from stationary phases cultures at time 0 h or at time 24 h (24 h incubation with moxifloxacin at 100 × MIC (6.4 mg/L) or at 100 mg/L. **(C)** Bacteria recovered from 24 h-biofilms (control at time 0) or after 24 h of incubation with moxifloxacin at 100 mg/L. The ordinate shows the flow cytometric profiles of the frequency of events (normalized to the mode value of the distribution) as a function of the fluorescence intensity (abscissa). Gray profiles: time 0 h; colored profiles: time 24 h.

We first compared the kinetics of activity of moxifloxacin at 100 mg/L against stationary phase cultures and biofilms of these two strains (Figure 5A). As previously described (Nguyen et al., 2020b), a plateau was observed for both strains after 5–10 h of incubation with moxifloxacin in stationary-phase cultures, with no further killing (up to 24 h), which is one of the key hallmarks of persisters (Balaban et al., 2004; Brauner et al., 2016). The percentage of persisters was approx. 0.01 and 0.04% of the initial population for the S-LP and the S-HP isolates, this difference being small but nevertheless statistically significant (*p*-value: 0.01; *t*-test). Flow cytometry profiles (Figure 5B) recorded at the beginning of the experiment (time 0) and after 24 h incubation with moxifloxacin at 100 × its MIC (6.4 mg/L) or at 100 mg/L showed only a minor decrease in fluorescence (minimal shift of the normalized fluorescence profile) for strain RN4220 (S-LP) and no decrease for strain 1214 (S-HP). Thus, there was only minimal or no dilution of the tracer, confirming that survivors only minimally divided or did not divide during the incubation time.

Moving now to data obtained with the same strains in biofilms, we see that killing occurred much more slowly (after a drop of approx. 1 log_10_ CFU occurring in about 1–2 h) than for bacteria in stationary phase culture, proceeding at a constant rate during the rest of the 24 h incubation period and without significant difference between the two strains (Figure 5A). Bacteria collected from 24 h-biofilms before addition of moxifloxacin (time 0) and after 24 h incubation with moxifloxacin were also examined in flow cytometry [Figure 5C; the GFP inducer was present during biofilm formation but removed at the time of addition of moxifloxacin (time 0)]. At time 0, most of the bacteria were highly fluorescent due to the maintenance of the inducer during biofilm growth. A partial dilution of the signal was nevertheless noted, especially in the S-HP isolate, which we attribute to a probable suboptimal penetration of the inducer in the growing biofilm. After 24 h incubation in the presence of 100 mg/L moxifloxacin, the fluorescence signal of the survivors was modestly reduced for the S-LP strain RN4220-pALc2084 and even more slightly for the S-HP strain 1214-pALc2084, indicating a limited division capacity in moxifloxacin-exposed biofilms, especially for the HP isolate.

The tolerant character of a strain toward an antibiotic can be estimated by the determination of the minimum duration of incubation with an antibiotic to achieve a predetermined extent of killing (MDK; Fridman et al., 2014). The table in Figure 5 shows that MDKs for a 90, 99, and 99.9% reduction of CFU in the presence of moxifloxacin at 100 mg/L were considerably shorter for bacteria in stationary cultures than for those in biofilms. In stationary cultures, MDK were short because they were calculated for reductions in CFU that still correspond to the elimination of the bulk of the population (preceding the plateau with infinitively long MDK against the persister subpopulation). In biofilms, MDKs were longer, demonstrating antibiotic tolerance even for small reductions in CFUs. In addition, the rate of killing as well as the reduction in CFUs reached at 24 h were similar for both strains.

## Discussion

In this work, we show that biofilm production by clinical isolates of *S. aureus* is variable and is in general higher in isolates that show a high relative persister fraction to moxifloxacin, as determined in stationary phase culture, and/or high MIC to this drug. The increase in matrix abundance is essentially due to polysaccharides, and is the main contributor to lack of activity for moxifloxacin against these biofilms. Yet, persistence and resistance are indirectly explaining these effects.

A first observation concerns the ability of clinical isolates to produce biofilm matrix depending on their persister or resistant character (Figure 1). Biofilms are typically enriched in dormant cells compared to planktonic cultures, sharing some similarity in this respect to stationary phase cultures (Spoering and Lewis, 2001). This phenotypic switch has been attributed to the impoverishment in nutrients and oxygen within the deepness of the biofilm structure (Conlon et al., 2015), which triggers a stringent response (Yan and Bassler, 2019) and promotes persister formation and antibiotic tolerance (Lewis, 2005; Nguyen et al., 2011). We reinforce this hypothesis by observing that isolates with high relative persister fractions produce a more abundant extracellular matrix than those with low relative persister fractions. This matrix is specifically enriched in polysaccharides, the amount of which is closely correlated with the level of expression of *icaA*, a gene from the operon coding for enzymes involved in PNAG synthesis (Figure 2). Importantly, PNAG has been shown to play a critical role in the adhesion of *S. aureus* to tissues (Lin et al., 2015) as well as in evasion from immune system (Cerca et al., 2007), contributing thus in its pathogenicity and persistence in the host. The expression of the *ica* operon is strictly controlled in staphylococci (Laverty et al., 2013; Arciola et al., 2015). Of interest, this regulation goes in parallel with that of persistence: the accessory-gene-regulator Agr impairs both the transcription of *icaA* (Cafiso et al., 2007) and persister formation (Xu et al., 2017) while the alternative sigma factor σ_*B*_ that increases *icaA* expression (Guldimann et al., 2016) is induced under stringent conditions (Reiss et al., 2012) and promotes persistence (Romilly et al., 2014). Previous works established higher biofilm production in antibiotic resistant *S. aureus* (Mahmoudi et al., 2019; Harika et al., 2020; Parastan et al., 2020; Senobar Tahaei et al., 2021), especially in those resistant to ciprofloxacin (Mahmoudi et al., 2019; Harika et al., 2020; Parastan et al., 2020), but none of these studies looked at the persister character of the isolates. To our knowledge, there is no established relationship between resistance to fluoroquinolones and the level of *icaA* expression, but well with the presence of the *icaADCB* operon (Manandhar et al., 2018). In addition, ciprofloxacin exposure in fluoroquinolone-resistant *S. aureus* promotes bacterial adhesion, which is the first step in biofilm formation, as part of the stress response it induces (Li et al., 2005). As the activation of stress response is a major trigger in persistence development (Nguyen et al., 2020b), we may therefore suggest that the correlation observed between moxifloxacin MIC and biomass is reflecting the inability to dissociate persistence and resistance for the majority of isolates, as stress activation in persisters promotes fluoroquinolone resistance (Nguyen et al., 2020b). A practical implication of this observation is that determining the persister character of clinical isolates in stationary phase culture could help predicting their ability to produce biofilm matrix. While the present demonstration is limited to moxifloxacin and *S. aureus*, it may already illustrate the concept that genes and proteins responsible for persistence in biofilms can be identified in planktonic populations, which are much easier to manipulate (Spoering and Lewis, 2001).

The second important but more expected observation is that the activity of moxifloxacin is noticeably low against biofilms (Figure 3). This has been previously reported by us and by others for a number of antibiotic classes and different bacterial species. But we document here that it applies to clinical isolates and that it results in an almost complete loss of effect of a highly bactericidal antibiotic toward the biomass, even though substantial killing of the bacteria can be obtained if markedly increasing its concentration. Two non-mutually exclusive hypotheses can be envisaged to explain this loss of activity, namely (i) a protective effect of the matrix hindering moxifloxacin diffusion within the biofilm, or (ii) the presence of antibiotic-tolerant dormant cells (Shapiro et al., 2011; Conlon, 2014; Butini et al., 2019).

Regarding the first hypothesis, we found that moxifloxacin activity in biofilms was significantly correlated with the amount of drug remaining in the biofilm after gentle washing, which was itself inversely correlated with the biofilm biomass, and more specifically with polysaccharide content (Figure 4). This observation is in line with previous data from our group (Siala et al., 2014). More specifically, the fact that moxifloxacin concentrations in biofilms falls below its MIC for resistant isolates (at the concentration used for confocal microscopy experiments) can contribute to explain the total lack of activity observed in these conditions. Fluoroquinolones like ciprofloxacin can adsorb on exopolysaccharides, with interactions notably demonstrated with hydroxyl groups (Zhang et al., 2018), which can reduce their availability in PNAG-rich biofilms. In this context, we know that agents disrupting the matrix also increase antibiotic activity against biofilm-embedded bacteria, with specific demonstrations made for fluoroquinolones and biofilms formed by staphylococci (Siala et al., 2014, 2016; Klinger-Strobel et al., 2017; Marques and Nelson, 2019). Biofilm matrix is, however, not an impassible barrier to antibiotics but rather acts by reducing their access to indwelling bacteria (Gristina et al., 1987; Zheng and Stewart, 2002; Jefferson et al., 2005; Siala et al., 2014). This would explain why the maximal relative efficacy (E**_*max*_**) of moxifloxacin against bacteria reaches similar values when tested against bacteria encased in biofilms produced by the reference strain ATCC 25923 or by the susceptible clinical isolates, provided its concentration is increased to match its decreased potency. Moreover, the dynamics of antibiotic penetration are influenced by the nature of the matrix (Zheng and Stewart, 2002; Stewart et al., 2009) and the spatial structure of the biofilm (Yan et al., 2018), which will create local antibiotic concentration gradients that may add selective pressure on the bacteria to enter a persister state (Waters et al., 2016).

This brings us to the second hypothesis, which rather considers dormant phenotypes as driving the poor antibiotic response in biofilms. A recent transcriptomic study suggests that persister cell formation may occur before biofilms reach maturity (Tomlinson et al., 2021). Here, we show (Figure 5) that the percentage of bacteria surviving to high concentrations of moxifloxacin is higher in biofilms than in stationary phase cultures, as previously suggested (Singh et al., 2009). Interestingly, both in biofilms and in broth, the initial rate of killing was high, as also recently observed for intracellular *S. aureus* when host cells are exposed to high concentrations of moxifloxacin (Peyrusson et al., 2020). In stationary phase cultures, this fast rate of killing eventually resulted in the almost complete elimination of the bacteria up to the point at which all survivors were persisters, incapable of division. In biofilms, the first phase of fast killing was quickly followed by a second, much slower phase suggestive of a tolerant phenotype, characterized by a limited ability to multiply. Thus, a significant proportion of cells within the biofilm remains sensitive to killing (Spoering and Lewis, 2001), provided sufficiently high concentrations of antibiotics and long enough times of exposure can be used.

Taken together, our observations suggest that antibiotic tolerance is not entirely dependent on the occurrence of these persisters, but is also critically dependent upon the production of large amounts of biomass. Our data thus support the concept that the poor responsiveness of bacteria to moxifloxacin within biofilms results from the combination of matrix effects and of slowed down multiplication rate, itself related to stress and linked to resistance. Of note, however, we also noticed that the persister character as determined in stationary phase culture is not predictive of the level of antibiotic-tolerance in biofilms, since MDK in biofilms are similar for strains that show low and/or high relative persister fractions in stationary phase cultures but produce biofilms with comparable biomass and CFU counts.

In a more clinically oriented perspective, our results highlight that, at clinically achievable concentrations, moxifloxacin is inactive against biofilms formed by clinical isolates, although fluoroquinolones are among the most active antibiotic classes against staphylococcal biofilms (Bauer et al., 2013; Siala et al., 2014, 2016; Masadeh et al., 2019). Therefore, strategies aiming at disrupting matrix integrity on the one hand, and at reviving dormant bacteria on the other hand, appear not only promising but should probably be developed together for optimum efficacy. These strategies, however, may stumble on difficulties related to reliance on results generated from simplified models of biofilm such as those we used here, where only exposure to a single antibiotic was considered, and where antibiotic concentrations were kept constant-over-time, ignoring the fluctuations in concentrations that are taking place in *in vivo* and could affect, positively as well as negatively, the bacterial response to the drug. The significance of our observations is also limited by the rarity of susceptible isolates with high-persister fractions identified in a larger screening (Nguyen et al., 2020a), which prevented us from including more isolates in the work. In addition, we cannot exclude that other characteristics of the isolates, not included in our analysis, may also affect biofilm formation or response to moxifloxacin in this collection.

In spite of these limitations, and if considering our data collectively, the most striking observation from this study remains that moxifloxacin activity depends on its local concentration in biofilm, which is reduced in most isolates with high-relative persister fractions due to matrix effects, and insufficient to kill resistant isolates due to their high MIC. Thus, our results tend to support a preponderant role of the matrix in impairing moxifloxacin activity against biofilms. Its abundance depends on the persister character of the strain (indissociable from resistance in the majority of the isolates), and prevents the drug to reach locally active concentrations when MICs are elevated.

## Data Availability Statement

The raw data supporting the conclusions of this article will be made available by the authors, without undue reservation.

## Author Contributions

TKN and FVB: conceptualization and writing—original draft preparation. TKN, FP, and WS: methodology and investigation. TKN, FP, WS, and FVB: formal analysis. NHP: resources. FP, HAN, and PMT: writing—review and editing. HAN, PMT, and FVB: supervision. FVB: funding acquisition. All authors contributed to the article and approved the submitted version.

## Conflict of Interest

The authors declare that the research was conducted in the absence of any commercial or financial relationships that could be construed as a potential conflict of interest.

## Publisher’s Note

All claims expressed in this article are solely those of the authors and do not necessarily represent those of their affiliated organizations, or those of the publisher, the editors and the reviewers. Any product that may be evaluated in this article, or claim that may be made by its manufacturer, is not guaranteed or endorsed by the publisher.

## Footnotes

1
https://eucast.org/clinical_breakpoints/
