# Supplementary material for: Activity of Moxifloxacin Against Biofilms Formed by Clinical Isolates of Staphylococcus aureus Differing by Their Resistant or Persister Character to Fluoroquinolones
Source: Front Microbiol. 2021 Dec 15;12:785573. doi: 10.3389/fmicb.2021.785573 (PMC8715871; doi:10.3389/fmicb.2021.785573)
Supplement: Supplementary file 1 [file Data_Sheet_1.PDF]

Supplementary Material

Activity of moxifloxacin against biofilms formed by clinical isolates of *Staphylococcus aureus* differing by their resistant or persister character to fluoroquinolones.

Tiep K. Nguyen, Frédéric Peyrusson, Wafi Siala, Nhung H. Pham, Hoang A. Nguyen, Paul M. Tulkens, Françoise Van Bambeke

Table S1: characteristics of the strains used in the present study (based on data published in (Nguyen et al., 2020a; Nguyen et al., 2020b).

Strain number	Origin[a]	Cefoxitine MIC (mg/L)	*mecA/mecC*[b]	Phenotype of resistance towards β-lactams	Moxifloxacin MIC (mg/L)[c]	Relative persister fraction to moxifloxacin[d]	Resulting phenotype
ATCC 25923	Reference		-/-	MSSA	0.032	1	
ATCC 33591	Reference		+/-	MRSA	0.032	1	
RN4220-pALc2084	(Nguyen et al., 2020b)			MSSA	0.064	8.0	Susceptible, low relative persister fraction (S-LP)
1214-pALc2084	(Nguyen et al., 2020b)			MSSA	0.064	26.7	Susceptible, high relative persister fraction (S-HP)
30549	Catheter	4	-/-	MSSA	0.032	0.8	Susceptible, low relative persister fraction (S-LP)
22782	Blood	32	+/-	MRSA	0.032	1.3	
30337	Catheter	16	+/-	MRSA	0.064	1.2	
69474	Catheter	8	+/-	MRSA	0.064	0.2	
69493	Pus	16	+/-	MRSA	0.064	1.2	
69687	Pus (catheter)	32	+/-	MRSA	0.25	1.0	
69783	Articulation	4	-/-	MSSA	0.032	37.2	Susceptible, high relative persister fraction (S-HP)
69505	Pus (tophi)	16	+/-	MRSA	0.032	45.5	
69867	Pus (tophi)	16	+/-	MRSA	0.064	74.8	
13890	Blood	4	-/-	MSSA	1	289.6	Resistant, high relative persister fraction (R-HP)
69519	Abscess	>32	+/-	MRSA	1	91.6	
69915	Pus (tophi)	16	+/-	MRSA	1	273.1	
30462	Catheter	4	-/-	MSSA	2	177.0	
35808	Blood	4	-/-	MSSA	2	29.0	
69781	Pus	4	-/-	MSSA	2	138.3	
34427	Pus (tophi)	4	-/-	MSSA	2	411.8	
35994	Articulation	4	-/-	MSSA	2	228.1	
25619	Blood	32	+/-	MRSA	2	111.3	

[a] as reported in medical files

[b] determined by PCR

[c] according to EUCAST susceptibility breakpoints, isolates MIC ≤ 0.25 mg/L are considered as susceptible to moxifloxacin

[d] calculated as the % of persisters after 5 h of incubation with 100 x MIC of moxifloxacin for each clinical isolate normalized to the % of persisters for ATCC 25923 in the same conditions. Isolates with relative persister fraction < 10 were considered as low-persisters and those with relative persister fraction > 10 as high-persisters.

Table S2: Pharmacodynamic parameters for concentration-response curves shown in Figure S4

strains	viability			biomass		
	Top[a] CFU/mL	Bottom[b] CFU/mL	EC_{50}[c] mg/L (CI)	Top[a] % control	Bottom[b] % control	EC_{50}[c] mg/L (CI)
ATCC 25923	6.6 ± 0.2 (A)	4.0 ± 0.2 (A)	0.9 (0.3-3.5) (A)	124 ± 4 (A)	34 ± 2 (A)	0.2 (0.1-0.3) (A)
Susceptible, low relative persister fraction (S-LP)	7.5 ± 0.1 (B)	5.0 ± 0.2 (B)	38.2 (18.0-80.9) (B)	111 ± 2 (A)	87 ± 3 (B)	3.7 (0.5-12.7) (B)
Susceptible, high relative persister fraction (S-HP)	7.5 ± 0.1 (B)	4.7 ± 0.2 (B)	25.4 (13.5-47.9) (B)	107 ± 4 (A)	90 ± 4 (B)	1 (0.003-40) (B)
Resistant, high relative persister fraction (R-HP)	7.8 ± 0.1 (B)	6.3 ± 0.3 (C)	345 (119-12158) (C)	100 ± 20 (A)	NA[d]	NA[d]

Pharmacodynamic parameters calculated based on a downward Hill-Langmuir function fitted to the data (changes in the number of viable bacteria (CFU/mL; in $\log_{10}$ units) or of crystal violet absorbance (% control value) compared to an untreated 24h-biofilm both after 24 h of incubation at 37°C

[a] value of the top plateau of the Hill equation.

[b] value of the bottom plateau of the Hill equation.

[c] moxifloxacin concentration for which the effect is halfway between E_{min} and E_{max}

[d] not applicable (Hill equation cannot be fitted to the data).

Statistical analysis: comparison between groups of strains for each parameter (one-way ANOVA with Tukey post-hoc test): data with different letters are significantly different from one another ($p < 0.05$). For EC_{50} values, statistics were performed on $\log_{10}$ values (symmetrically distributed). Data are shown as means and SD (E_{max}) or as mean and 95% confidence interval (EC_{50}).

Figure S1 Quantification of biofilms made by clinical isolates as compared to the reference strains ATCC 25923 and ATCC 33591 after 48 h of incubation in TGN (with renewal of the medium at 24 h). Crystal violet (CV) absorbance (A) and CFUs counts (B) are shown for the whole population of clinical isolates *vs*. the reference strain ATCC 25923 (black dot) or ATCC33591 (gray dot). Symbols for clinical isolates depend on their resistant or persister phenotype (S-LP [red]: susceptible, with low relative persister fraction; S-HP [green] susceptible, with high relative persister fraction; R-HP [blue]: resistant, with high relative persister fraction). Data are shown as mean ± SD of 6 independent experiments for the references, and as mean ± SD for clinical isolates, with each dot representing the mean value of 2-3 independent experiments for each isolate. Statistical analysis: A-B: Mann-Whitney test comparing clinical isolates with ATCC25923 (black) or ATCC33591 (gray): ****: $p < 0.0001$; ** $p < 0.01$.

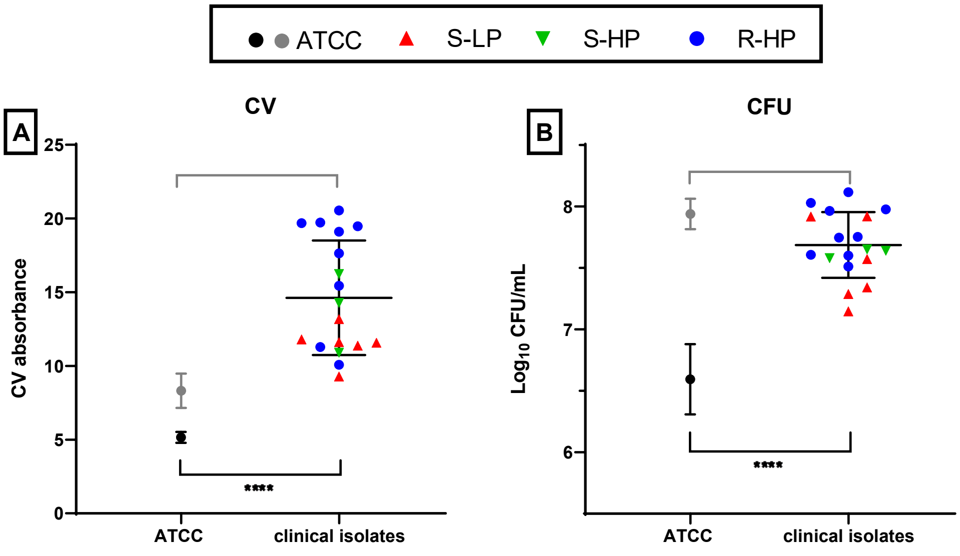

Figure S2. Quantification of metabolic activity in clinical isolates as compared to a reference strain (ATCC 25923) in planktonic cultures at an OD_{620nm} of 1 and incubated during 1h with 10 mg/L resazurin in PBS. A: fluorescence signal emitted by resorufin, the fluorescent metabolite of resazurin, for the whole population of clinical isolates *vs*. the reference strain ATCC 25923 (black dot) and for clinical isolates (S-LP [red]: susceptible, with low relative persister fraction; S-HP [green] susceptible, with high relative persister fraction; R-HP [blue]: resistant, with high relative persister fraction). Data are shown as mean ± SD of 6 independent experiments for ATCC 25923, and as mean ± SD of the different isolates for clinical isolates, with each dot representing the mean value of 2 independent experiments for each isolate. B: correlation between resorufin fluorescence and the relative persister fraction. Statistical analysis: A: Mann-Whitney test: *: $p < 0.05$; B: r (correction coefficient) and p-values for the correlation.

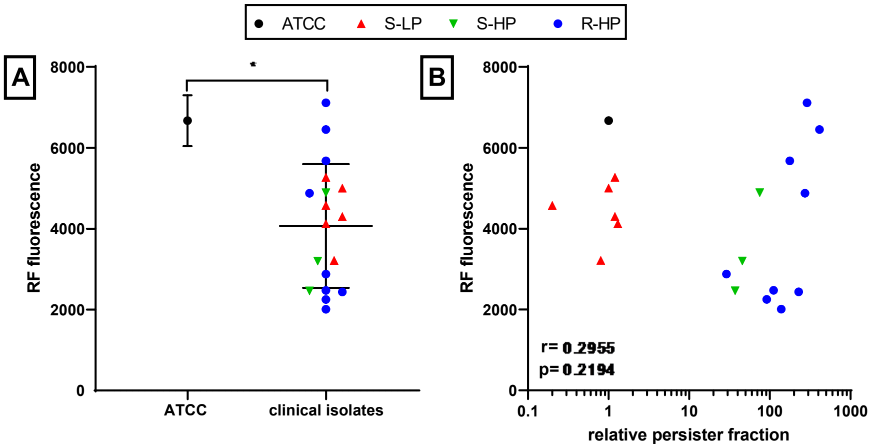

Figure S3. Quantification of proteins (A) and eDNA (B) content in biofilms made by clinical isolates (S-LP [red]: susceptible, with low relative persister fraction; S-HP [green] susceptible, with high relative persister fraction; R-HP [blue]: resistant, with high relative persister fraction) and as compared to reference strains ATCC 25923 (black dot) and ATCC33591 (gray dot). The graphs show the correlations between proteins or eDNA contents in 48 h biofilms and crystal violet absorbance. Each dot representing the mean value of 3 independent experiments for each isolate. Statistical analysis: r (correction coefficient) and p-values for the correlation.

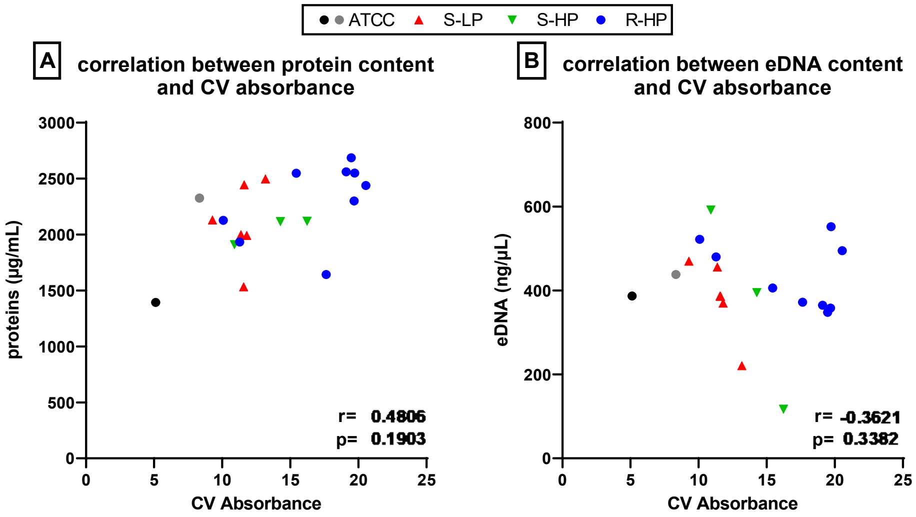

Figure S4. Concentration-response curves of the activity of moxifloxacin (MXF) against 24h-biofilms formed by clinical isolates as compared to a reference strain (ATCC 25923), after 24 h of incubation with the antibiotic over a broad range of concentrations. Panels A-C: Log_{10} of CFU numbers. The data were used to fit sigmoidal function (Hill equation), for ATCC 25923 (black lines) and pooled values from all clinical isolates (colored lines), respectively; they are shown as means and SD (n=6) for ATCC 25923 and as mean (n=2) for each individual isolate. The dotted lines above and below the regression line show the 95% confidence interval. Panel A: susceptible isolates with low relative persister fraction [S-LP]; panel B: susceptible isolates with high relative persister fraction [S-HP]; panel C: resistant isolates with high relative persister fraction [R-HP]. ATCC 25923 is represented in all graphs to facilitate comparisons.

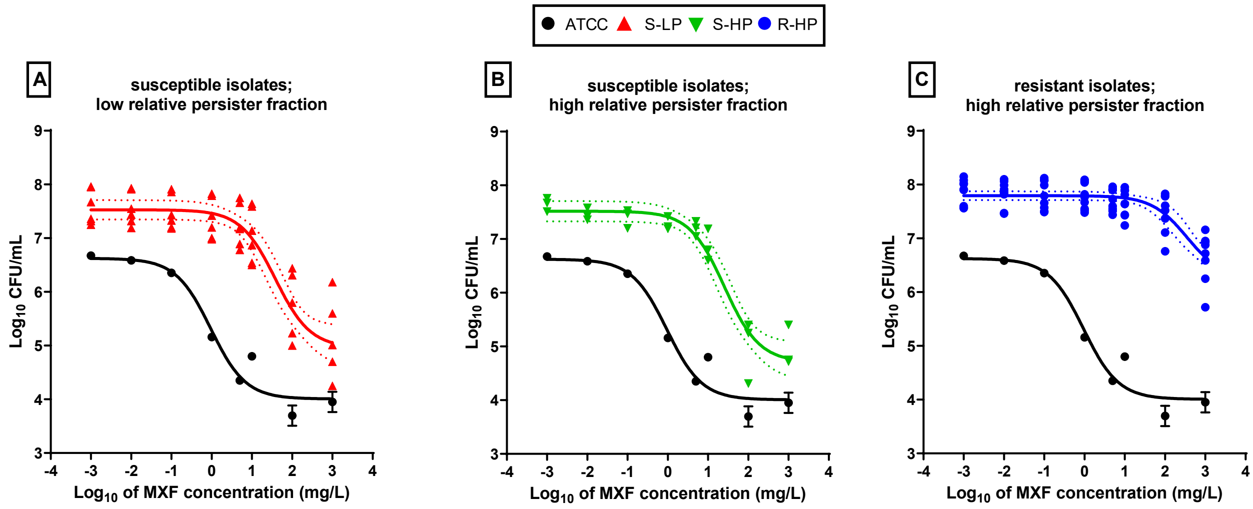

References

Nguyen, T. K., Argudin, M. A., Deplano, A., Pham, N. H., Nguyen, H. A., Tulkens, P. M., Dodemont, M., and Van Bambeke, F. (2020a). Antibiotic resistance, biofilm formation, and intracellular survival as possible determinants of persistent or recurrent infections by Staphylococcus aureus in a Vietnamese tertiary hospital. Focus on bacterial response to moxifloxacin. *Microb. Drug Resist.* 26, 537-544.

Nguyen, T. K., Peyrusson, F., Dodemont, M., Pham, N. H., Nguyen, H. A., Tulkens, P. M., and Van Bambeke, F. (2020b). The persister character of clinical isolates of Staphylococcus aureus contributes to faster evolution to resistance and higher survival in THP-1 monocytes: a study with moxifloxacin. *Front Microbiol.* 11, 587364.
